# Case Report of a 23‐year‐old first gravida presenting with breathlessness and oxygen saturation of 80%

**DOI:** 10.1002/ccr3.3756

**Published:** 2021-02-02

**Authors:** Raphael Porsche, René Höhn, Kirsten Krüger, Johannes Kalbhenn, Joachim Bansbach

**Affiliations:** ^1^ Department of Anesthesiology and Critical Care Faculty of Medicine Medical Center‐University of Freiburg University of Freiburg Freiburg Germany; ^2^ Department of Congenital Heart Defects and Pediatric Cardiology Faculty of Medicine Medical Center‐University of Freiburg University of Freiburg Freiburg Germany; ^3^ Department of Medicine III (Interdisciplinary Medical Intensive Care) Faculty of Medicine Medical Center‐University of Freiburg University of Freiburg Freiburg Germany

**Keywords:** Eisenmenger, interdisciplinary cooperation, pregnancy, pulmonary hypertension

## Abstract

Central principles are interdisciplinary teamwork and keeping the ratio between pulmonary vascular resistance (PVR) and systemic vascular resistance (SVR) balanced to avoid vicious circle with right heart failure and hypoxia.

## Graphical abstract

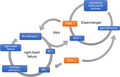

## 1 INTRODUCTION

A case of a 23‐year‐old first gravida presenting with Eisenmenger. Cesarean section was performed under epidural anesthesia due to maternal indication. Central principle is keeping the ratio between pulmonary vascular resistance (PVR) and systemic vascular resistance (SVR) balanced to avoid vicious circle with right heart failure and hypoxia.

The experience with diagnosis and treatment of VSD with Eisenmenger´s reaction in adults is almost lost in developed countries as most of the patients are diagnosed before birth or in the early childhood and therapy is initiated early. The wave of refugees from poor and third world countries brings these conditions back to our hospitals and forces us to take them into account and to refresh our knowledge about seldom pathologies. For the clinician, the question arises which diagnostic methods are most suitable for these patients and safest for mother and unborn child. Understanding the pathophysiology that leads to a vicious circle of right heart failure and hypoxia is essential for the treatment of these patients. This manuscript adheres to the applicable EQUATOR guideline.

## 2 CASE DESCRIPTION

A 23‐year‐old first gravida in the 28th week of pregnancy presented to a general hospital in Germany with premature labor. She was a refugee from Kosovo, and communication was impeded by a language barrier. Before pregnancy, she stated, she had been fully resilient. Because of breathlessness with peripheral oxygen saturation of 80%, she was admitted to intensive care unit (ICU). With high‐flow nasal oxygen therapy (HFNOT) by inspired oxygen concentration of 60% saturation did not exceed 92%. On examination, she presented watch glass nails which she had since childhood. Her father showed an unclear heart disease and her twin died under the birth.

Auscultation of the lung was normal, and pneumothorax or pleural effusion was excluded by sonography. Out of consideration for the unborn, the responsible physician decided against X‐ray or computed tomography. The patient had no fever, and laboratory findings were unsuspicious for infection. Electrocardiography showed a sinus rhythm with a rightward axis deviation.

A cardiac vitium was suspected but could not be proofed. Echocardiography showed right ventricular hypertrophy with pulmonary hypertension (PAPsys 85 mm Hg). Due to equalization of pressure between right and left ventricle, no ventricular septal defect could be detected in Color Doppler Echocardiography.

Therapeutic anticoagulation with unfractionated heparin was started by suspected pulmonary embolism. Tocolysis with Atosiban and lung maturity with Betamethason were performed. Due to the high birth risk, the patient was transferred to our university tertiary referral medical center. Duplex sonography showed no evidence of deep leg vein thrombosis, and D‐dimers were low. Based on these findings and hypertrophy of the right ventricle, acute right heart strain due to pulmonary embolism appeared unlikely.

In interdisciplinary consensus between gynecologists, cardiologists, neonatologists, anesthesiologists, and intensive care physicians, indication for an immediate cesarean section was given from maternal indication.

After establishing basis monitoring, an epidural catheter was inserted at lumbar 3‐4 level in loss of resistance technique. A cumulative dose of 112.5 mg of ropivacaine was given in graded aliquots to achieve an anesthesia level of T 6. During section, administration of inhaled nitric monoxide (iNO) with 30 parts per million (ppm) via a CPAP‐mask was applied. The patient delivered a female child with 1190 g. APGAR was 5/4/7. After Cesarean section, the patient received further treatment on the intensive care unit.

**FIGURE 1:**
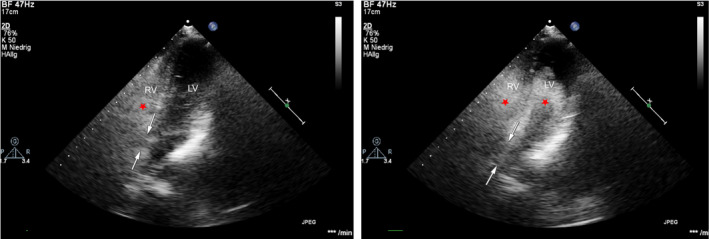
Exemplary image from apical transthoracic echocardiography, bubble test (red star) shows a perimembranous VSD (indicated by white arrows, 12 × 14 mm) with Eisenmenger reaction (RV—right ventricle; LV—left ventricle)

Echocardiography by a specialist for congenital heart defects confirmed the preoperative findings but did not immediately reveal a cardiac vitium. The examiner instructed the patient to perform a Valsalva maneuver. Now a strong contrast could be detected in the left ventricle. Focussing on this area of the septum further sonography revealed a high perimembranous ventricular septal defect (12 × 14 mm) with Eisenmenger syndrome (Figure 1). Computed tomography of the chest showed a normal pulmonary status and computed cardiac tomography confirmed a large, high ventricular septal defect with shunt between the left and right ventricular outflow tract and a strongly dilated pulmonary trunk. Treatment of pulmonary hypertension with sildenafil and bosentan was started. On day 7, the patient was brought to a normal ward in good general condition.

## 3 DISCUSSION

This case underlines that treatment of such patients should take place in clinical centers with an interdisciplinary team containing experts for cardiology, congenital heart defects, anesthesiology, gynecology, neonatology, and intensive care. The extend of the patient's cardiac condition was unclear at first. Transthoracic echocardiograms described severe pulmonary arterial hypertension (PAH) with suspicion of a VSD. After ruling out pulmonary embolism (PE) as a cause for PAH, we discussed the necessity for further PAH diagnostics before delivery. We decided neither to perform computed tomography because of radiation potentially harming the unborn, nor right heart catheterization because of risk of arrhythmias. Even though there is no evidence of this, the accepted maximum limit of radiation exposure to the fetus during pregnancy is a cumulative dose of 50 mSv. Chest X‐ray causes a radiation load of only 0.3 mSv, but significance is low. Cardiovascular Computed Tomography (CCT) is much more informative, but high radiation doses are required (~8 mSv). Echocardiography and Cardiovascular Magnetic Resonance (CMR) seem to be safe during pregnancy and not associated with adverse effects on the fetus. Problem of the CMR is that the patient must be able to lie flat for at least 30 minutes. For most patients with breathlessness, this is not possible, so echocardiography was diagnostic tool of choice. Focus was to evaluate the best moment for delivery. The child, being at 28 weeks gestation, was at risk of prematurity but lung maturity already was induced. The mothers clinical state was deteriorating over time and physical stress reliably led to clinical decompensation with oxygen desaturations. We prioritized the survival of the mother and we wanted to avoid an emergency delivery situation with everyone not being optimally prepared. Cesarean section is described as the method of choice in patients with PAH and/or Eisenmenger.^1^ Although there are case reports of successfully using general anesthesia in this case,^2^ usually epidural anesthesia is performed. Epidural was our anesthesia method of choice, because of the possibility to tightly control the rate of induction and effect on sympatholysis, systemic vascular resistance (SVR), mean arterial pressure (MAP), and hearth rate (HR).^3^ In addition, positive pressure ventilation with risk of right heart decompensation could be avoided.

Epidural anesthesia was established preoperatively at intensive care unit to reduce stress levels for the patient. We chose against a Swan‐Ganz catheter because of the risk of arrhythmias, although measurement of pulmonary artery pressure would have been useful. Eisenmenger patients have an increased risk for severe Swan‐Ganz catheter‐related complications through paradoxical embolism as described in case reports.^4^

**FIGURE 2:**
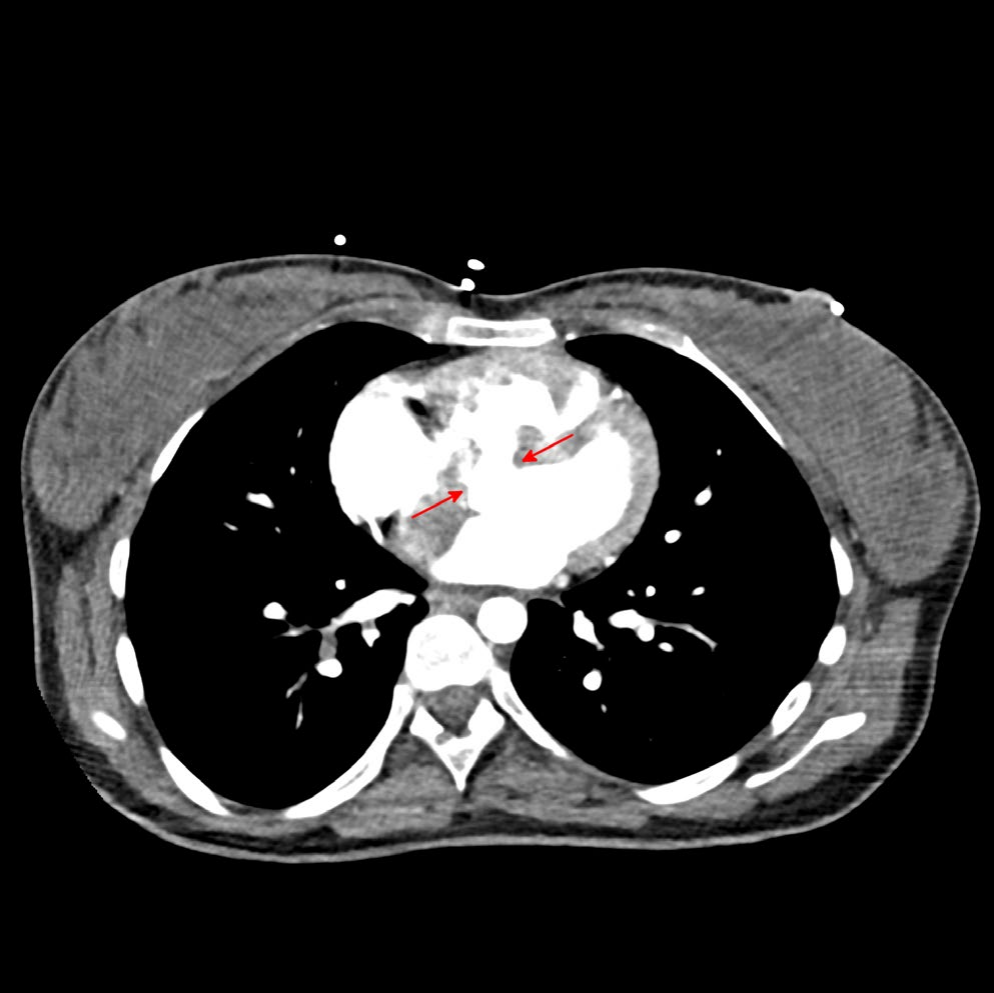
Exemplary transversal image from contrast medium enhanced thoracic computed tomography; large ventricular septal defect with shunt between the left and right ventricular outflow tract indicated by red arrows

Rescue plan in case of cardiac decompensation was applying inhaled nitric oxide trough a CPAP‐mask and crashing to either veno‐venous or even veno‐arterial extracorporeal membrane oxygenation (ECMO). After delivery, we initiated a triple PAH‐treatment with inhaled iloprost (prostacyclin receptor antagonist), oral sildenafil (phosphodiesterase type‐5 inhibitor), and oral bosentan (endothelin receptor antagonist).^1^ Iloprost had to be stopped because of epistaxis. We could also complement diagnostics by computer tomography which proved the existence of a ventricular septal defect with shunt reversal which guided our further approach (Figure 2).

**FIGURE 3:**
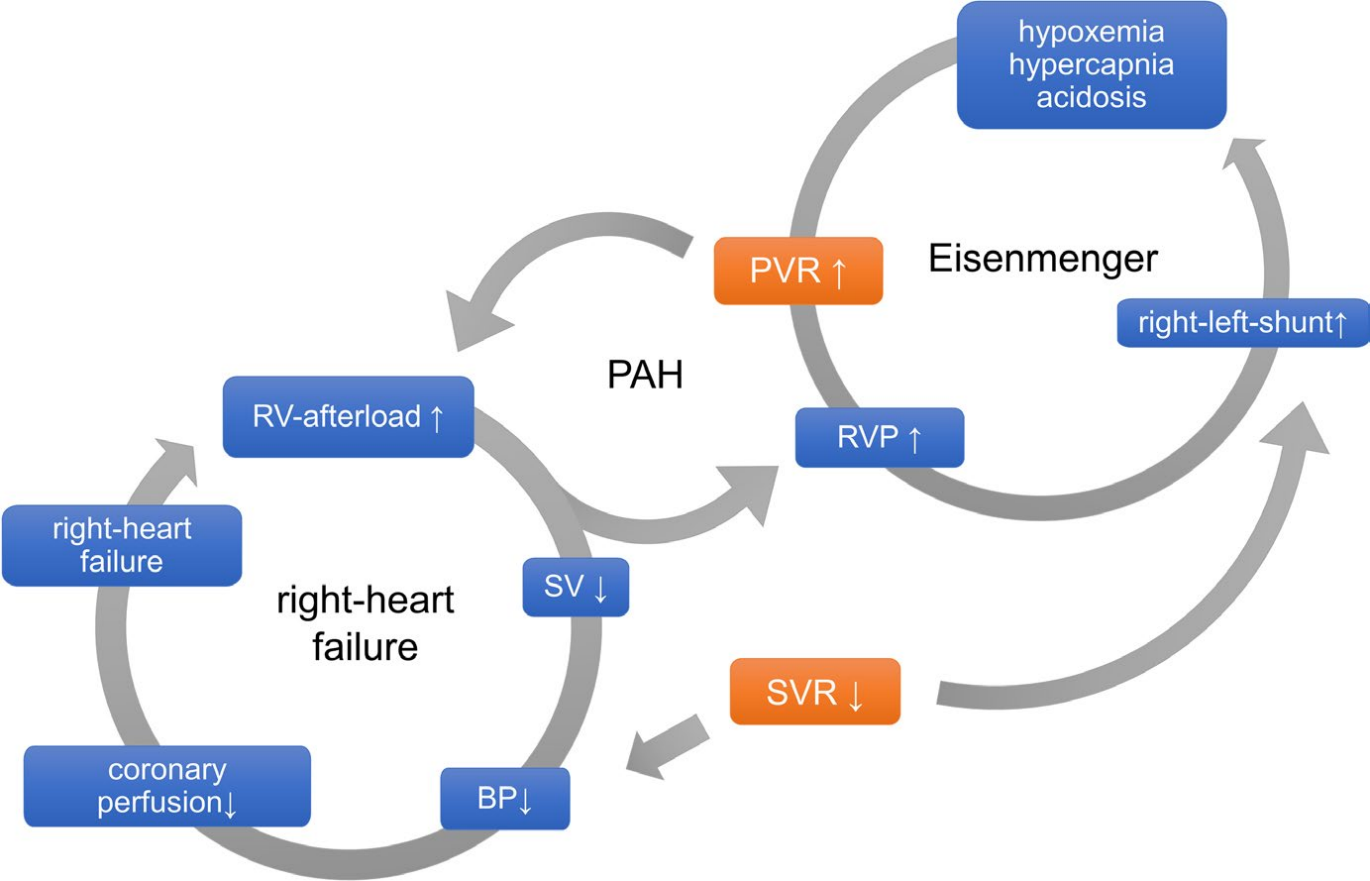
Schematic illustration of the right‐left cardiac hypoxemic shunt (Eisenmenger) and right heart failure vicious circles by ventricular septal defect. Increasing pulmonary vascular resistance (PVR) by stress, positive pressure ventilation, coughing, hypoxemia, hypercapnia, sedation or decrease of systemic vascular resistance (SVR) by hypnotics, vasodilators, sepsis etc results in worsening of Eisenmenger´s phenomenon. PAH—pulmonary arterial hypertension; SV—stroke volume; BP—blood pressure; RV—right ventricle

VSD in this patient leads to left‐right shunt in childhood. Pulmonary arteries were chronically overflooded and consecutive hyperplasia of the vascular muscularis followed. Caused by the increased pulmonal vascular resistance, the right ventricle became hypertrophic. Eisenmenger´s reaction is the result of these mechanisms finally leading to right‐left shunt when either pulmonary vascular resistance (PVR) rises or systemic vascular resistance (SVR) is lowered. Central principles in treatment of these patients are constantly keeping the ratio between PVR and SVR balanced. Increasing PVR (stress, positive pressure ventilation, coughing, hypoxemia, hypercapnia, and sedation) or decreasing SVR (hypnotics and vasodilators) results in worsening of the right‐left cardiac hypoxemic shunt (Eisenmenger) and right heart failure vicious circles (Figure 3). Since pregnancy leads to a physiologic decrease in SVR by 20%‐30%,^5^ usually the clinical state worsens over the course of pregnancy.^6^ Peripartum maternal mortality of Eisenmenger patients is high (20%‐50%) and therefore avoiding pregnancy and early termination of pregnancy usually is advised.^7^ Our escalation levels of treating decompensations included calming the patient, applying oxygen to reduce PVR, knees to chest position to increase SVR, applying inhaled NO through CPAP, and giving vasopressin. Vasopressin was the vasopressor of choice because its minimal effect on PVR, its positive effect on PVR/SVR ratio, and its potential pulmonary vasodilatory effect.^8^, ^9^ Causal treatment at this stage of disease was only possible by mechanically occluding the VSD in combination with lung transplantation. Lung transplantation is needed since situations with increased right ventricular afterload in absence of the VSD as a pressure relief valve could lead to right heart failure. The patient was educated about the importance of regular medical checkups and pharmacotherapy. Also, further pregnancies should be avoided.

## DISCLOSURE

The legal representative of the patient gave his consent for publishing the case report and the images.

## CONFLICT OF INTEREST

None.

## AUTHOR CONTRIBUTIONS

Raphael Porsche: wrote the manuscript and created the graphics. René Höhn: helped to revise the manuscript. Kirsten Krüger: helped to revise the manuscript. Johannes Kalbhenn: revised the manuscript. Joachim Bansbach: wrote and revised the manuscript.

## ETHICS APPROVAL AND CONSENT TO PARTICIPATE

The patient gave her written consent for the scientific processing and publication of her treatment case.
